# Spatial-Frequency–Dependent Contrast Sensitivity in Early and Intermediate Age-Related Macular Degeneration

**DOI:** 10.1016/j.xops.2026.101351

**Published:** 2026-08-04

**Authors:** Stefan Futterknecht, Maximilian Pfau, Ursula Hall, Chrysoula Gabrani, Philipp Anders, Georg Ansari, Ghislaine L. Traber, Hendrik P. Scholl, Nicolas Feltgen, Michael Herzog, Simona A. Garobbio, Kristina Pfau

**Affiliations:** 1 Institute of Molecular and Clinical Ophthalmology Basel, Basel, Switzerland; 2 Department of Ophthalmology, Universitätsspital Basel, Basel, Switzerland; 3 Department of Ophthalmology, University of Bonn, Bonn, Germany; 4 Vista Augenklinik, Binningen, Switzerland; 5 Department of Clinical Pharmacology, Medizinische Universität Wien, Vienna, Austria; 6 Pallas Kliniken AG, Olten, Switzerland; 7 European Vision Institute, Basel, Switzerland; 8 Laboratory of Psychophysics, Ecole Polytechnique Federale de Lausanne, Lausanne, Switzerland

**Keywords:** Contrast sensitivity, Age-related macular degeneration, Outcome measure, Clinical trials

## Abstract

**Objective:**

To evaluate spatial-frequency–dependent differences in contrast sensitivity across early and intermediate age-related macular degeneration (eAMD and iAMD) and explore the discriminative value of visual function metrics beyond age.

**Design:**

Cross-sectional observational study.

**Subjects:**

Ninety-six participants from the Multimodal Assessment of Visual Function in Intermediate AMD study, including 49 controls, 14 participants with eAMD, and 33 with iAMD.

**Methods:**

Contrast sensitivity was assessed using the quick contrast sensitivity function method at six spatial frequencies from 1 to 18 cycles per degree, with derivation of contrast acuity and area under the log contrast sensitivity function (AULCSF). Additional visual function measures included microperimetry mean sensitivity, best-corrected visual acuity, and low-luminance visual acuity. Linear mixed-effects models evaluated associations between AMD stage, spatial frequency, and contrast sensitivity after adjustment for age- and lens-related covariates. Secondary analyses assessed the discriminative performance of visual function metrics using receiver operating characteristic analyses.

**Main Outcome Measures:**

Contrast sensitivity across spatial frequencies and discriminative performance of visual function metrics.

**Results:**

In age- and lens-adjusted mixed-effects models, contrast sensitivity differed significantly by AMD stage (*P* < 0.001), with a significant AMD stage × spatial frequency interaction (*P* = 0.035). Compared with controls, iAMD showed lower adjusted contrast sensitivity across most spatial frequencies, whereas eAMD differences were most pronounced at intermediate spatial frequencies. Discriminative analyses showed stronger separation of controls from iAMD than from eAMD. Microperimetry mean sensitivity provided the highest numerical discrimination for control versus iAMD and eAMD versus iAMD, whereas high-frequency contrast sensitivity and AULCSF performed best for control versus eAMD. Incremental gains beyond age- and lens-related covariates were modest for eAMD but larger for iAMD and for distinguishing eAMD from iAMD.

**Conclusions:**

Contrast sensitivity differences in AMD are spatial-frequency–dependent and vary by disease stage after adjustment for age and lens-related covariates. Quick contrast sensitivity function–derived metrics showed modest incremental discrimination for eAMD, whereas microperimetry mean sensitivity and selected low-frequency contrast sensitivity measures better distinguished iAMD from controls and eAMD. These findings support spatial-frequency–resolved functional testing as an exploratory measure for AMD characterization; larger, age- and lens-balanced longitudinal cohorts are needed to determine prognostic utility.

**Financial Disclosure(s):**

Proprietary or commercial disclosure may be found in the Footnotes and Disclosures at the end of this article.

Age-related macular degeneration (AMD) is a leading cause of irreversible visual impairment in individuals aged 50 years and older, affecting an estimated 200 million people worldwide.^1^^,^^2^ With aging populations, the global burden of AMD is projected to rise, underscoring the need for timely diagnosis and effective intervention.^1^ While late-stage AMD is often marked by profound visual loss, early and intermediate forms are typically asymptomatic or present with only subtle functional changes—yet these stages offer the greatest opportunity for intervention and prevention of progression.^3^^,^^4^

Detecting functional deficits in early AMD (eAMD) remains a clinical challenge. Traditional assessments such as best-corrected visual acuity (BCVA), though widely used in trials and practice, are poorly suited to capturing the nuanced functional changes characteristic of nonadvanced disease.^5^^,^^6^ Even low-luminance visual acuity, designed to unmask subclinical deficits under mesopic conditions, has shown limited sensitivity in early stages.^7^^,^^8^ This discrepancy allows individuals with eAMD or intermediate AMD (iAMD) to maintain normal visual acuity, despite significant dysfunction at the photoreceptor and retinal pigment epithelium (RPE) level—posing a major challenge for early detection and inclusion in clinical trials targeting disease modification at its earliest stages.^9^^,^^10^

Rod-mediated dark adaptation testing is a sensitive functional measure for eAMD, capable of detecting subtle photoreceptor and RPE dysfunction before structural changes are visible on funduscopy.^11^^,^^12^ However, its broader clinical adoption is limited by practical challenges: the test is time-consuming, requires sustained patient cooperation, and depends on specialized equipment that is not widely available outside research settings.

Microperimetry provides spatially resolved sensitivity maps of the macula, enabling detection of localized deficits that precede overt visual loss.^13^^,^^14^ Emerging functional assays such as the quick contrast sensitivity function (qCSF) test also offer the potential to address these limitations. The qCSF method offers a rapid, reliable measure of contrast sensitivity (CS) across a range of spatial frequencies, yielding granular insights into visual function not captured by conventional acuity testing.^15^, ^16^, ^17^

In this study, we aimed to (1) characterize spatial-frequency–dependent differences in CS across early and iAMD using age- and lens-adjusted mixed-effects models and (2) evaluate the incremental discriminative value of CS and other visual function metrics using regression-based receiver operating characteristic analyses. By integrating spatial-frequency–resolved modeling with age-adjusted diagnostic analyses, we sought to provide a more nuanced assessment of functional alterations in AMD and their potential relevance for disease staging and future longitudinal studies.

## Methods

### Study Design

This cross-sectional analysis was conducted as part of the baseline assessment of the ongoing longitudinal Multimodal Assessment of Visual Function in Intermediate AMD study. The study was designed to investigate the diagnostic performance of a comprehensive battery of visual function tests in AMD. All participants provided written informed consent, and the study adhered to the tenets of the Declaration of Helsinki with ethical approval obtained from the local ethics committee (Ethikkommission Nordwest- und Zentralschweiz).

### Participants

Participants were enrolled from the retina clinics at the University Hospital Basel and through a combination of community outreach initiatives and referrals from private ophthalmologists. The study included individuals spanning the early to intermediate stages of AMD, as well as control participants without AMD. Based on clinical evaluation and multimodal imaging, participants were classified into three groups according to the Beckman classification^3^: controls (individuals without any drusen or pigmentary abnormalities associated with AMD), eAMD (characterized by the presence of medium-sized drusen between 63 and 125 μm without accompanying AMD pigmentary abnormalities), and iAMD (defined by the presence of large drusen (>125 μm) and/or any AMD-associated pigmentary abnormalities). Participants exhibiting signs of late-stage AMD (i.e., neovascular AMD or any geographic atrophy) were excluded from this study.

Eligibility criteria included an age of 50 years or older, adequate cognitive ability to perform the visual function test battery, and sufficient ocular media clarity to ensure reliable imaging and testing. Individuals were excluded if they showed evidence of advanced AMD (geographic atrophy or choroidal neovascularization), had other retinal or optic nerve diseases, or exhibited significant cognitive impairment (Montreal Cognitive Assessment score < 20). For controls, the nondominant eye was selected unless contraindicated, to ensure consistent testing conditions across groups.

### Visual Function Tests

All participants underwent a standardized battery of visual function tests in both eyes, encompassing traditional acuity assessments, CS testing, microperimetry, and selected psychophysical tasks. Testing was performed under standardized room illumination, and best refractive correction was applied throughout. Analyses were restricted to 1 study eye per participant; for controls, the nondominant eye was selected, and for AMD participants, the eye meeting the respective AMD classification criteria was used.

Microperimetry was conducted under mesopic lighting conditions using the MAIA microperimeter (CenterVue). The tested grid comprised 24 points covering the central 10 degrees of the macula. Stimuli corresponded to Goldmann size III (0.43° visual angle) and were presented over a background luminance of approximately 1.3 cd/m^2^, with a maximum stimulus luminance of 318.3 cd/m^2^ and a dynamic range of 0 to 36 dB. Thresholds were determined using a 4–2 staircase procedure. Mean sensitivity, defined as the average differential light sensitivity across the tested macular locations, was used as the principal microperimetric outcome measure.

Contrast sensitivity was evaluated using the Manifold Platform (Adaptive Sensory Technology), which presents Sloan letter triplets across a wide range of contrast levels and spatial frequencies (1–27 cycles per degree [cpd]). Testing was performed under standardized mesopic room lighting (∼7 lux). For each eye, CS thresholds were extracted at six predefined spatial frequencies (1, 1.5, 3, 6, 12, and 18 cpd). From these, two composite metrics were derived: the area under the log contrast sensitivity function (AULCSF), summarizing global visual performance, and contrast acuity, indicating the highest spatial frequency detectable at maximum contrast.

Traditional visual acuity metrics were also assessed. Best-corrected visual acuity was measured under photopic conditions using standardized ETDRS charts at 4 m. Low-luminance visual acuity was assessed by repeating BCVA measurements with a 2.0-log unit neutral density filter, maintaining chart luminance at 130 cd/m^2^ to simulate mesopic viewing conditions.

To complement these standardized clinical tests, participants also completed a custom psychophysical test battery administered on a calibrated liquid-crystal display screen at 2 m. Gabor CS testing required participants to discriminate the orientation of briefly presented (200 ms), 3 cpd Gabor stimuli under mesopic illumination. The QUEST adaptive staircase algorithm was used to determine contrast thresholds corresponding to 75% correct responses.

This multimodal approach was designed to comprehensively capture functional impairments across the visual system, particularly those that precede overt anatomical changes. Special emphasis was placed on microperimetry and qCSF testing due to their documented sensitivity to early photoreceptor and RPE dysfunction in AMD.

### Statistical Analysis

Descriptive statistics were calculated for demographic and clinical variables. Continuous variables were compared across groups using one-way analysis of variance or Kruskal–Wallis tests, and categorical variables using Fisher exact tests. Visual function metrics were analyzed on the standardized modified z-score scale.

The primary analysis evaluated CS as a function of AMD group and spatial frequency using a linear mixed-effects model. The model included AMD group, spatial frequency, and their interaction as fixed effects; age, lens status (phakic vs. pseudophakic), and mild cataract status as covariates; and a participant-level random intercept. Spatial frequency was modeled as a categorical variable to avoid imposing assumptions about the functional form of the qCSF and to enable frequency-specific group comparisons. This approach accounted for repeated measurements across spatial frequencies within individuals and enabled assessment of spatial-frequency–dependent differences between AMD stages.

Type III analysis of variance with Satterthwaite approximation was used to evaluate model effects. Estimated marginal means were calculated to derive adjusted CS profiles and pairwise group contrasts across spatial frequencies. *P* values for pairwise comparisons were adjusted using the Benjamini–Hochberg method.

Secondary discriminative analyses used logistic regression models for predefined binary group comparisons: controls versus any AMD, controls versus eAMD, controls versus iAMD, and eAMD versus iAMD. For each comparison, an age-only model was first fitted, followed by models including age and 1 visual function metric. Receiver operating characteristic curves were generated from predicted probabilities, and area under the curve (AUC) values with 95% confidence intervals (CIs) were estimated using nonparametric bootstrap resampling with 2000 iterations. Incremental value beyond age was quantified as the difference in AUC (ΔAUC) between the age-only model and the age plus marker model and was assessed using likelihood-ratio tests. To evaluate lens-related confounding, additional models were fitted including age, lens status, and mild cataract as baseline covariates. Incremental value of visual function metrics beyond this extended baseline model was assessed using ΔAUC and likelihood-ratio tests.

Given the limited sample size and correlation among visual function metrics, no multivariable model-selection procedure was performed. No internal optimism correction, nested cross-validation, or external validation was performed; therefore, receiver operating characteristic (ROC) analyses were considered exploratory. No formal statistical comparisons of AUC values were performed.

All analyses were performed in R version 4.4.2 using packages including lme4, lmerTest, emmeans, pROC, boot, ggplot2, and gt.

## Results

### Baseline Characteristics

A total of 96 participants were included, comprising 49 controls, 14 participants with eAMD, and 33 with iAMD. Age differed significantly across groups, with higher age in eAMD and iAMD than controls (65.4 ± 8.5, 72.1 ± 6.7, and 74.1 ± 7.1 years, respectively; *P* < 0.001). Body mass index, smoking history, Montreal Cognitive Assessment score, sex, race, smoking status, lens status, glaucoma, and diabetes did not differ significantly across groups. Mild cataract was more frequent in eAMD and iAMD than in controls (16.3%, 42.9%, and 57.6%, respectively; *P* < 0.001; Table 1).

**Table 1:** Baseline Demographic and Clinical Characteristics by AMD Group

Variable	Control (*n* = 49)	eAMD (*n* = 14)	iAMD (*n* = 33)	*P* Value
Age [years]	65.4 ± 8.5	72.1 ± 6.7	74.1 ± 7.1	<0.001
BMI [kg/m^2^]	24.9 ± 3.6	26.4 ± 4.0	26.7 ± 5.2	0.172
Smoking years	9.3 ± 14.3	10.1 ± 12.3	15.5 ± 17.2	0.176
MoCA	26.4 ± 2.5	25.6 ± 2.7	25.9 ± 2.6	0.527
Gender: female	30 (61.2%)	6 (42.9%)	18 (54.5%)	0.458
Gender: male	19 (38.8%)	8 (57.1%)	15 (45.5%)	0.458
Race: White	49 (100%)	14 (100%)	32 (97%)	0.490
Race: other	0 (0%)	0 (0%)	1 (3%)	0.490
Smoker: no	29 (59.2%)	6 (42.9%)	13 (39.4%)	0.157
Smoker: past	14 (28.6%)	8 (57.1%)	16 (48.5%)	0.157
Smoker: current	6 (12.2%)	0 (0%)	4 (12.1%)	0.157
Phakic	45 (91.8%)	11 (78.6%)	26 (78.8%)	0.136
Pseudophakic	4 (8.2%)	3 (21.4%)	7 (21.2%)	0.136
Mild cataract	8 (16.3%)	6 (42.9%)	19 (57.6%)	<0.001
Glaucoma	0 (0%)	0 (0%)	2 (6.1%)	0.237
Diabetes	1 (2%)	0 (0%)	0 (0%)	1.000

AMD = age-related macular degeneration; BMI = body mass index; MoCA = Montreal Cognitive Assessment.

Continuous variables are presented as mean ± standard deviation; categorical variables are presented as number and percentage. Group comparisons were performed using Kruskal–Wallis tests for continuous variables and Fisher exact tests for categorical variables. AMD groups were categorized as control, early AMD (eAMD), and intermediate AMD (iAMD) according to Beckman classification criteria.

### Visual Function Tests

**Figure 1:**
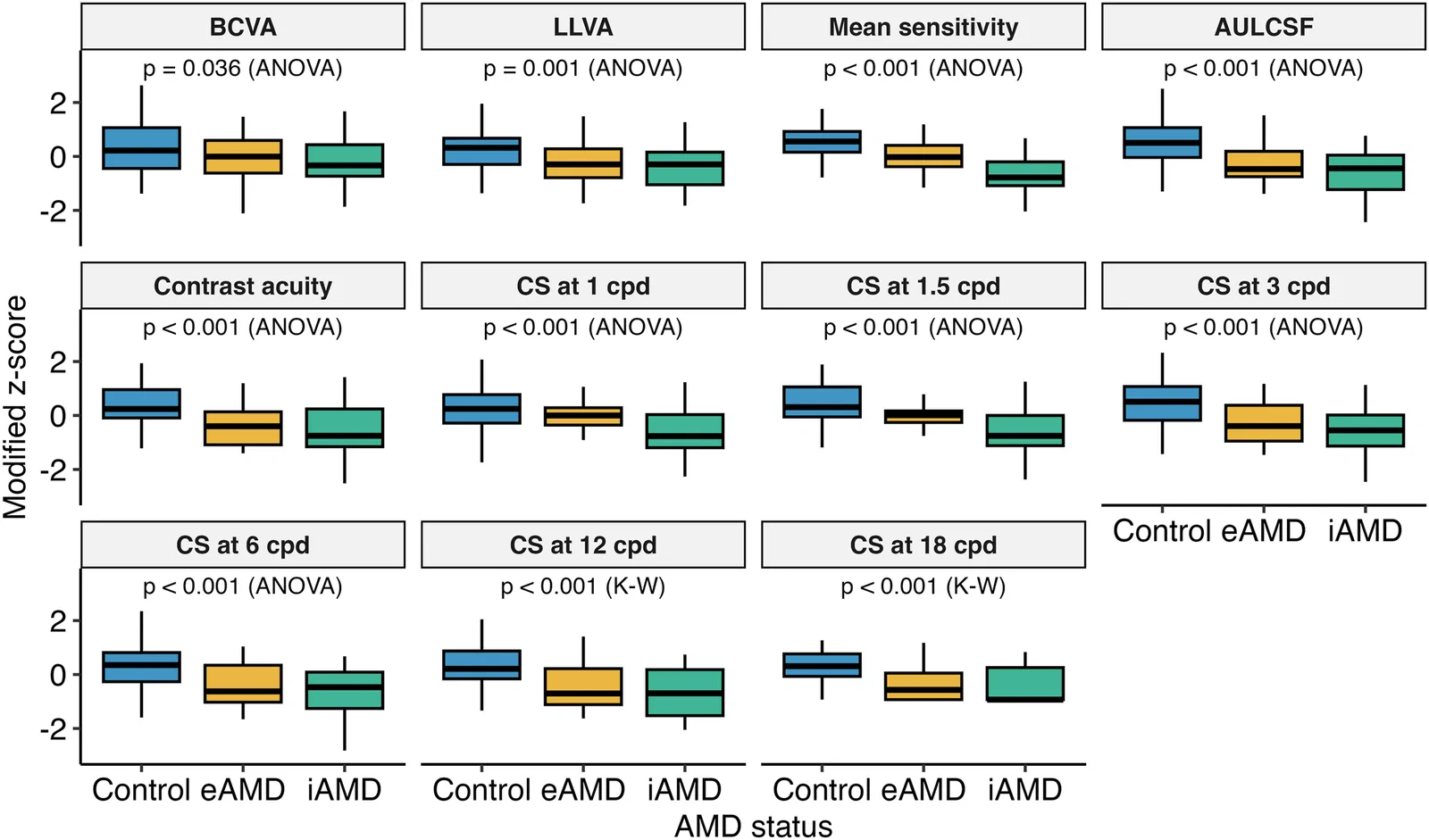
Distribution of visual function metrics by AMD group. Boxplots display the distribution of standardized visual function metrics across control, early AMD (eAMD), and intermediate AMD (iAMD) groups. All metrics were standardized to modified z-scores. Statistical comparisons were conducted using one-way ANOVA or Kruskal–Wallis (K–W) tests, as appropriate. *P* values were adjusted for multiple comparisons using the Benjamini–Hochberg procedure to control the false discovery rate. Adjusted *P* values are indicated above each plot. Group differences were observed for several metrics; however, these unadjusted comparisons do not account for potential confounding by age- or lens-related factors and should be interpreted descriptively. AMD = age-related macular degeneration; ANOVA = analysis of variance.

Unadjusted visual function metrics varied across AMD stages (Fig 1). These analyses are presented descriptively and do not account for potential confounding by age or lens-related factors. Therefore, subsequent analyses focused on age- and lens-adjusted modeling of CS and on exploratory discriminative analyses.

### Spatial-frequency–dependent Differences in CS

**Figure 2:**
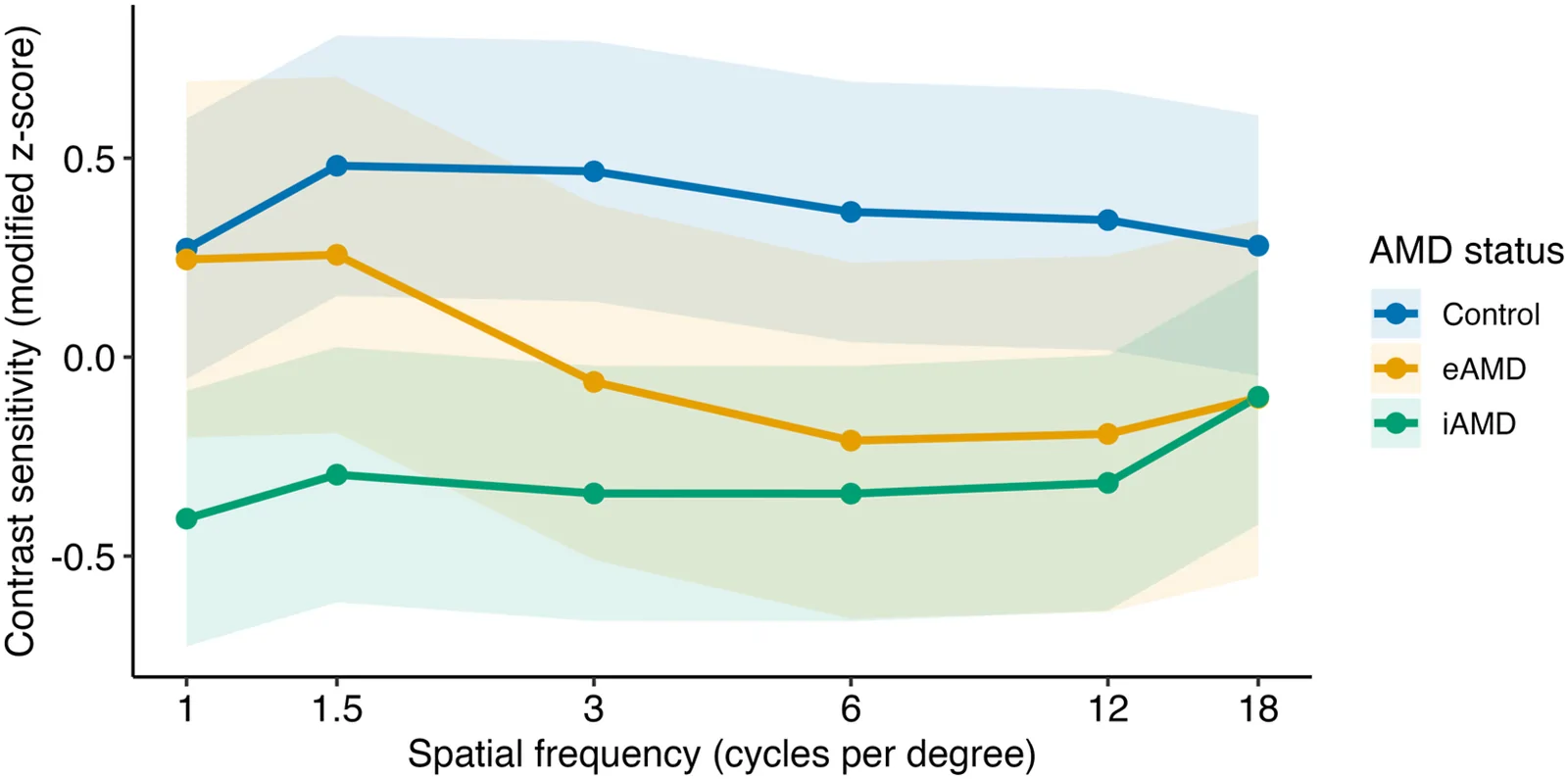
Age- and lens-adjusted contrast sensitivity function profiles across AMD stages. Model-based estimated marginal means of contrast sensitivity (CS) are shown across spatial frequencies from 1 to 18 cycles per degree (cpd) for controls, eAMD, and iAMD. Estimates were derived from a linear mixed-effects model including AMD stage, spatial frequency, their interaction, age, lens status, mild cataract, and a participant-level random intercept. Shaded ribbons indicate 95% confidence intervals. Contrast sensitivity differed by AMD stage, and the AMD stage × spatial frequency interaction was significant, indicating spatial-frequency–dependent group differences. AMD = age-related macular degeneration; cpd = cycles per degree; eAMD = early AMD; iAMD = intermediate AMD.

In the primary age- and lens-adjusted linear mixed-effects model, CS differed significantly by AMD stage (F_2,88_ = 7.94, *P* < 0.001), and the AMD stage × spatial frequency interaction was significant (F_10,455_ = 1.96, *P* = 0.035), indicating that group differences varied across spatial frequencies (Fig 2; Supplementary Table 1, available at www.ophthalmologyscience.org). Age was independently associated with CS (F_1,88_ = 19.64, *P* < 0.001). Lens status showed a nonsignificant trend (F_1,88_ = 2.99, *P* = 0.087), whereas mild cataract was not associated with CS (F_1,88_ = 0.10, *P* = 0.749).

In pairwise contrasts, iAMD showed significantly lower CS than controls at 1, 1.5, 3, 6, and 12 cpd after Benjamini–Hochberg adjustment, whereas the difference at 18 cpd was not significant (Supplementary Table 2, available at www.ophthalmologyscience.org). Differences between controls and eAMD were smaller and were significant at 6 and 12 cpd, whereas the comparison at 3 cpd was borderline significant (*P* = 0.050). Comparisons between eAMD and iAMD were significant at 1 and 1.5 cpd, suggesting greater low-frequency impairment in iAMD.

### Age-Adjusted Discriminative Performance

**Figure 3:**
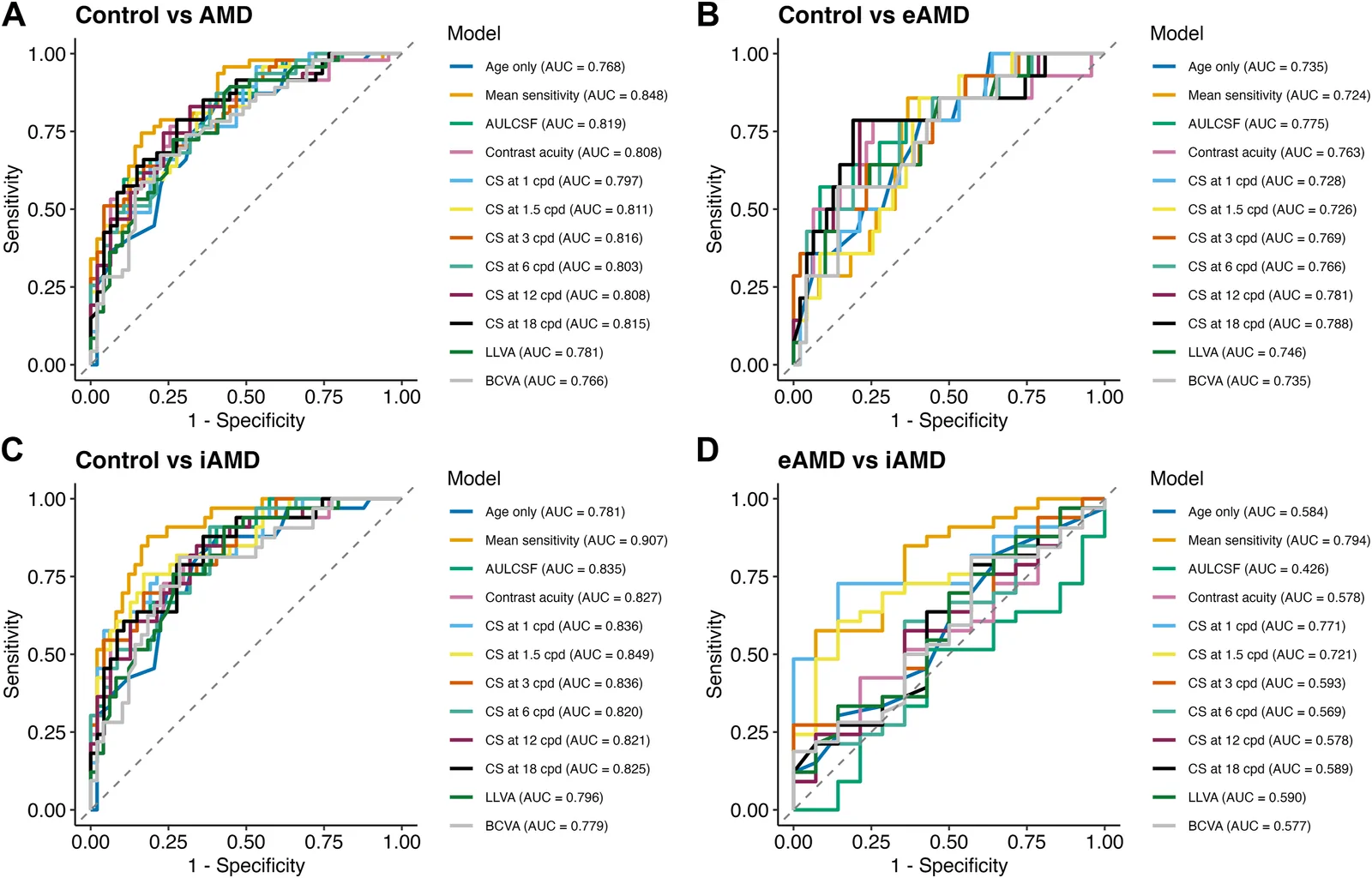
Receiver operating characteristic (ROC) curves for age-adjusted visual function metrics across binary group comparisons. Receiver operating characteristic curves show the discriminative performance of age-only and age plus visual function logistic regression models for four predefined binary comparisons: **(A)** controls versus any AMD, **(B)** controls versus eAMD, **(C)** controls versus iAMD, and **(D)** eAMD versus iAMD. Curves were generated from predicted probabilities of logistic regression models. AUC values are shown in the figure legends; 95% confidence intervals are provided in Supplementary Table 3. AMD = age-related macular degeneration; AUC = area under the receiver operating characteristic curve; eAMD = early AMD; iAMD = intermediate AMD.

In secondary ROC analyses, age alone showed moderate discrimination across comparisons, with AUCs ranging from 0.584 for eAMD versus iAMD to 0.781 for controls versus iAMD (Fig 3; Supplementary Table 3, available at www.ophthalmologyscience.org). For controls versus eAMD, the numerically highest age-adjusted AUCs were observed for CS at 18 cpd (AUC, 0.788; 95% CI, 0.636–0.940), CS at 12 cpd (AUC, 0.781; 95% CI, 0.631–0.932), and AULCSF (AUC, 0.775; 95% CI, 0.626–0.924). However, CIs were wide and overlapping.

For controls versus iAMD, mean sensitivity showed the highest point estimates of AUC (AUC, 0.907; 95% CI, 0.844–0.969), followed by several contrast sensitivity function (CSF)-derived metrics with AUCs exceeding 0.80. For eAMD versus iAMD, microperimetry mean sensitivity (AUC, 0.794; 95% CI, 0.653–0.936) and CS at 1 cpd (AUC, 0.771; 95% CI, 0.636–0.905) showed the highest numerical discrimination.

### Incremental Discriminative Value Beyond Age

In exploratory single-marker logistic regression analyses, the addition of individual visual function metrics to age-only models produced modest numerical improvements in discriminative performance (Supplementary Table 4, available at www.ophthalmologyscience.org). In the control versus eAMD comparison, the largest numerical increase in AUC was observed for CS at 18 cpd (AUC increased from 0.732 to 0.788; ΔAUC = 0.056; likelihood ratio *P* = 0.039), followed by CS at 12 cpd (ΔAUC = 0.049; *P* = 0.051), AULCSF (ΔAUC = 0.043; *P* = 0.039), CS at 3 cpd (ΔAUC = 0.037; *P* = 0.045), and CS at 6 cpd (ΔAUC = 0.034; *P* = 0.038). Low-frequency CS, BCVA, low-luminance visual acuity, and microperimetry mean sensitivity did not, or only minimally, improve discriminative performance beyond age in this comparison.

In the control versus iAMD comparison, microperimetry mean sensitivity showed the largest incremental improvement beyond age (ΔAUC = 0.125; likelihood ratio *P* < 0.001). Smaller improvements were observed for CS at 1.5 cpd (ΔAUC = 0.072; *P* < 0.001), 3 cpd (ΔAUC = 0.059; *P* < 0.001), 1 cpd (ΔAUC = 0.059; *P* = 0.001), and AULCSF (ΔAUC = 0.058; *P* < 0.001).

In the eAMD versus iAMD comparison, microperimetry mean sensitivity demonstrated the largest incremental improvement beyond age (ΔAUC = 0.210; likelihood ratio *P* < 0.001), followed by CS at 1 cpd (ΔAUC = 0.186; *P* = 0.005) and CS at 1.5 cpd (ΔAUC = 0.136; *P* = 0.019). Most remaining visual function metrics showed minimal or no incremental discriminative value beyond age. Because these analyses were exploratory and several visual function metrics were correlated, results should be interpreted descriptively.

### Lens-Adjusted Incremental Analyses

After additional adjustment for lens status and mild cataract, incremental discrimination for controls versus eAMD was attenuated (Supplementary Table 5, available at www.ophthalmologyscience.org). The largest ΔAUC values were observed for AULCSF (ΔAUC = 0.033; likelihood ratio *P* = 0.068), CS at 18 cpd (ΔAUC = 0.030; *P* = 0.051), and CS at 12 cpd (ΔAUC = 0.029; *P* = 0.073), none of which reached statistical significance. In controls versus iAMD, mean sensitivity showed the largest incremental metric beyond age and lens covariates (ΔAUC = 0.090; *P* < 0.001). In eAMD versus iAMD, mean sensitivity, CS at 1 cpd, and CS at 1.5 cpd retained the largest incremental values.

## Discussion

In this cross-sectional analysis of the Multimodal Assessment of Visual Function in Intermediate AMD cohort, CS differed across AMD stages in a spatial-frequency–dependent manner after adjustment for age- and lens-related factors. The significant AMD stage × spatial frequency interaction indicates that functional impairment in AMD is not uniform across the CSF, but varies across spatial scales. While secondary discriminative analyses showed that the incremental value of individual qCSF-derived metrics beyond age was modest for eAMD, particularly after lens adjustment, the persistence of spatial-frequency–dependent differences after multivariable adjustment supports the presence of disease-related functional alterations beyond aging alone. These findings are consistent with prior reports demonstrating functional abnormalities in AMD that overlap partially with age-related visual decline.^5^^,^^6^^,^^13^

In iAMD, CS reductions were evident across a broad range of spatial frequencies, consistent with more global functional impairment. In eAMD, differences relative to controls were smaller and associated with substantial uncertainty, reflecting both limited sample size and overlapping functional distributions. Nevertheless, deficits were most apparent at intermediate spatial frequencies. Mechanistically, greater impairment at higher spatial frequencies may reflect early vulnerability of central photoreceptor pathways and cone-rich retinal regions implicated in AMD pathophysiology.^9^^,^^10^

Importantly, the present analyses extend prior work in the same cohort by modeling the CSF as a whole rather than emphasizing isolated spatial frequencies.^17^ By incorporating a group × spatial frequency interaction within an age- and lens-adjusted mixed-effects framework, the study accounts for repeated within-subject measurements across the CSF and reduces reliance on isolated point estimates from correlated qCSF-derived parameters. The persistence of significant group differences after adjustment for age- and lens-related covariates suggests that these functional alterations are not explained solely by aging- or lens-related factors. In addition, our findings complement prior studies demonstrating associations between functional impairment, retinal sensitivity loss, and structural AMD features.^5^^,^^7^^,^^13^^,^^14^^,^^18^

In exploratory ROC analyses, several qCSF-derived metrics yielded numerically higher discriminative performance than age alone, particularly for control versus eAMD comparisons. However, improvements beyond age were relatively small and associated with wide, overlapping CIs, reflecting the limited sample size of the eAMD subgroup. Additional adjustment for lens status and mild cataract further attenuated these effects, underscoring the importance of accounting for optical confounders when evaluating high-spatial-frequency visual function in aging populations. Taken together, these findings suggest that qCSF-derived measures may provide complementary functional information beyond demographic risk factors, although their independent cross-sectional discriminative value for eAMD remains limited in the present cohort.

Despite these limitations, spatial-frequency–resolved CS testing remains an attractive approach for functional phenotyping in AMD. The qCSF method enables rapid estimation of the full CSF with good repeatability and substantially greater functional granularity than conventional acuity testing.^15^, ^16^, ^17^ Rather than serving as standalone diagnostic measures, qCSF-derived metrics may be most valuable as complementary functional endpoints in longitudinal studies, multimodal risk models, or structure–function analyses integrating retinal imaging biomarkers.^4^^,^^19^, ^20^, ^21^, ^22^ In this context, spatial-frequency–specific functional testing may help refine characterization of early retinal dysfunction before overt visual acuity loss occurs.

Several limitations should be considered. The small number of participants with eAMD (n = 14) limits statistical power and contributes to imprecision in subgroup analyses. The cross-sectional design precludes assessment of disease progression or prognostic value. Although age- and lens-related factors were explicitly modeled, residual confounding cannot be excluded. Furthermore, ROC analyses were exploratory and were not internally or externally validated. Finally, the single-center design may limit generalizability.

In conclusion, CS in AMD is spatial-frequency–dependent and varies by disease stage after adjustment for age- and lens-related factors. Spatial-frequency–resolved qCSF testing detected functional alterations that persisted after multivariable adjustment, supporting its potential utility for functional characterization of early and iAMD. Although the incremental cross-sectional discriminative value beyond age was modest in this cohort, these findings support further investigation of qCSF-derived measures as complementary functional endpoints in larger longitudinal and multimodal AMD studies.

## Declaration of Generative AI and AI-Assisted Technologies in the Writing Process

During the preparation of this work, the authors used ChatGPT (OpenAI) for language editing. After using this tool/service, the authors reviewed and edited the content as needed and take full responsibility for the content of the publication.
