# Supplementary material for: Spatial-Frequency–Dependent Contrast Sensitivity in Early and Intermediate Age-Related Macular Degeneration
Source: Ophthalmol Sci. 2026 Aug 4;6(10):101351. doi: 10.1016/j.xops.2026.101351 (PMC13595137; doi:10.1016/j.xops.2026.101351)
Supplement: Supplementary file 1 [file mmc1.pdf]

Supplementary Table 1

Model term	Sum of squares	Mean square	Numerator df	Denominator df	F statistic	P value
AMD stage	4.22	2.11	2	88.00	7.94	<0.001
Spatial frequency	2.12	0.42	5	455.00	1.60	0.159
Age	5.21	5.21	1	88.00	19.64	<0.001
Lens status	0.80	0.80	1	88.00	2.99	0.087
Mild cataract	0.03	0.03	1	88.00	0.10	0.749
AMD stage × spatial frequency	5.22	0.52	10	455.00	1.96	0.035

Supplementary Table 1. Type III ANOVA for the age- and lens-adjusted contrast sensitivity function model.

Type III analysis of variance was performed using Satterthwaite approximation for denominator degrees of freedom. The linear mixed-effects model included AMD stage, spatial frequency, their interaction, age, lens status, mild cataract, and a participant-level random intercept. AMD, age-related macular degeneration.
