# Supplementary material for: Spatial-Frequency–Dependent Contrast Sensitivity in Early and Intermediate Age-Related Macular Degeneration
Source: Ophthalmol Sci. 2026 Aug 4;6(10):101351. doi: 10.1016/j.xops.2026.101351 (PMC13595137; doi:10.1016/j.xops.2026.101351)
Supplement: Supplementary file 2 [file mmc2.pdf]

Supplementary Table 2

Spatial frequency, cpd	Contrast	Adjusted mean difference	95% CI	BH-adjusted P value
1	Control vs eAMD	0.027	-0.460 to 0.515	0.913
1	Control vs iAMD	0.678	0.285 to 1.072	0.002
1	eAMD vs iAMD	0.651	0.157 to 1.146	0.015
1.5	Control vs eAMD	0.224	-0.264 to 0.711	0.366
1.5	Control vs iAMD	0.776	0.383 to 1.169	<0.001
1.5	eAMD vs iAMD	0.552	0.058 to 1.047	0.043
3	Control vs eAMD	0.529	0.041 to 1.017	0.050
3	Control vs iAMD	0.809	0.416 to 1.203	<0.001
3	eAMD vs iAMD	0.280	-0.214 to 0.775	0.265
6	Control vs eAMD	0.575	0.087 to 1.062	0.032
6	Control vs iAMD	0.708	0.315 to 1.101	0.001
6	eAMD vs iAMD	0.133	-0.361 to 0.628	0.595
12	Control vs eAMD	0.537	0.050 to 1.025	0.046
12	Control vs iAMD	0.660	0.267 to 1.053	0.003
12	eAMD vs iAMD	0.123	-0.371 to 0.617	0.624
18	Control vs eAMD	0.383	-0.105 to 0.870	0.185
18	Control vs iAMD	0.380	-0.013 to 0.773	0.174
18	eAMD vs iAMD	-0.003	-0.497 to 0.492	0.991

Supplementary Table 2. Age- and lens-adjusted group differences in contrast sensitivity by spatial frequency.

Pairwise group contrasts were estimated from the linear mixed-effects model using estimated marginal means. Confidence intervals are unadjusted 95% confidence intervals. P values were adjusted using the Benjamini–Hochberg method. Positive estimates indicate higher adjusted CS

in the first group than in the second group. AMD, age-related macular degeneration; BH, Benjamini–Hochberg; cpd, cycles per degree; CS, contrast sensitivity.
