# Supplementary material for: Spatial-Frequency–Dependent Contrast Sensitivity in Early and Intermediate Age-Related Macular Degeneration
Source: Ophthalmol Sci. 2026 Aug 4;6(10):101351. doi: 10.1016/j.xops.2026.101351 (PMC13595137; doi:10.1016/j.xops.2026.101351)
Supplement: Supplementary file 3 [file mmc3.pdf]

Supplementary Table 3

Metric	N	Cases	AUC (95% CI)	Threshold (Youden index)	Sensitivity / Specificity
**Control vs AMD**					
Mean sensitivity	96	47	0.848 [0.772–0.925]	0.58	0.74 / 0.84
AULCSF	94	47	0.819 [0.736–0.902]	0.63	0.60 / 0.89
CS at 3 cpd	94	47	0.816 [0.733–0.899]	0.61	0.64 / 0.83
CS at 18 cpd	94	47	0.815 [0.729–0.900]	0.53	0.79 / 0.72
CS at 1.5 cpd	94	47	0.811 [0.726–0.895]	0.46	0.81 / 0.66
Contrast acuity	94	47	0.808 [0.720–0.896]	0.49	0.79 / 0.72
CS at 12 cpd	94	47	0.808 [0.721–0.894]	0.48	0.83 / 0.68
CS at 6 cpd	94	47	0.803 [0.717–0.890]	0.40	0.87 / 0.60
CS at 1 cpd	94	47	0.797 [0.709–0.885]	0.59	0.66 / 0.79
LLVA	96	47	0.781 [0.690–0.872]	0.52	0.72 / 0.73
Age only	96	47	0.768 [0.674–0.862]	0.39	0.85 / 0.59
BCVA	95	46	0.766 [0.671–0.861]	0.57	0.67 / 0.78
**Control vs eAMD**					
CS at 18 cpd	61	14	0.788 [0.636–0.940]	0.31	0.79 / 0.81
CS at 12 cpd	61	14	0.781 [0.631–0.932]	0.28	0.79 / 0.79
AULCSF	61	14	0.775 [0.626–0.924]	0.38	0.57 / 0.91
CS at 3 cpd	61	14	0.769 [0.625–0.913]	0.31	0.64 / 0.77
CS at 6 cpd	61	14	0.766 [0.620–0.912]	0.25	0.79 / 0.66
Contrast acuity	61	14	0.763 [0.597–0.928]	0.26	0.79 / 0.74
LLVA	63	14	0.746 [0.601–0.892]	0.34	0.57 / 0.86
BCVA	63	14	0.735 [0.591–0.879]	0.34	0.57 / 0.86
Age only	63	14	0.735 [0.597–0.874]	0.18	0.79 / 0.59
CS at 1 cpd	61	14	0.728 [0.587–0.869]	0.20	0.79 / 0.62
CS at 1.5 cpd	61	14	0.726 [0.588–0.865]	0.20	0.86 / 0.60
Mean sensitivity	63	14	0.724 [0.586–0.863]	0.19	0.86 / 0.63
**Control vs iAMD**					
Mean sensitivity	82	33	0.907 [0.844–0.969]	0.36	0.88 / 0.82
CS at 1.5 cpd	80	33	0.849 [0.764–0.934]	0.51	0.76 / 0.83
CS at 3 cpd	80	33	0.836 [0.749–0.922]	0.51	0.70 / 0.83
CS at 1 cpd	80	33	0.836 [0.746–0.925]	0.46	0.76 / 0.79
AULCSF	80	33	0.835 [0.749–0.920]	0.27	0.91 / 0.60
Contrast acuity	80	33	0.827 [0.736–0.918]	0.34	0.88 / 0.64

CS at 18 cpd	80	33	0.825 [0.733–0.916]	0.33	0.88 / 0.64
CS at 12 cpd	80	33	0.821 [0.730–0.913]	0.36	0.85 / 0.66
CS at 6 cpd	80	33	0.820 [0.730–0.909]	0.31	0.91 / 0.62
LLVA	82	33	0.796 [0.700–0.892]	0.42	0.76 / 0.73
Age only	82	33	0.781 [0.680–0.882]	0.36	0.82 / 0.65
BCVA	81	32	0.779 [0.675–0.883]	0.40	0.81 / 0.71
**eAMD vs iAMD**					
Mean sensitivity	47	33	0.794 [0.653–0.936]	0.82	0.58 / 0.93
CS at 1 cpd	47	33	0.771 [0.636–0.905]	0.73	0.73 / 0.86
CS at 1.5 cpd	47	33	0.721 [0.570–0.871]	0.75	0.61 / 0.86
CS at 3 cpd	47	33	0.593 [0.414–0.772]	0.78	0.27 / 1.00
LLVA	47	33	0.590 [0.406–0.773]	0.70	0.70 / 0.50
CS at 18 cpd	47	33	0.589 [0.405–0.773]	0.67	0.79 / 0.43
Age only	47	33	0.584 [0.403–0.766]	0.69	0.70 / 0.50
Contrast acuity	47	33	0.578 [0.401–0.755]	0.73	0.42 / 0.79
CS at 12 cpd	47	33	0.578 [0.395–0.761]	0.71	0.58 / 0.64
BCVA	46	32	0.577 [0.395–0.759]	0.67	0.81 / 0.43
CS at 6 cpd	47	33	0.569 [0.383–0.756]	0.70	0.61 / 0.64
AULCSF	47	33	0.426 [0.247–0.606]	0.71	0.52 / 0.57

Supplementary Table 3. Discriminative performance of age-only and age-plus-marker models across AMD groups.

AUCs were estimated from logistic regression models including age alone or age plus the respective visual function metric. Sensitivity and specificity were calculated at the Youden-index threshold. Analyses are shown for controls versus any AMD, controls versus eAMD, controls versus iAMD, and eAMD versus iAMD. Values should be interpreted descriptively because several visual function metrics are correlated and confidence intervals overlap. AMD, age-related macular degeneration; AUC, area under the receiver operating characteristic curve; AULCSF, area under the log contrast sensitivity function; BCVA, best-corrected visual acuity; CI, confidence interval; cpd, cycles per degree; CS, contrast sensitivity; LLVA, low-luminance visual acuity.
