# Supplementary material for: Spatial-Frequency–Dependent Contrast Sensitivity in Early and Intermediate Age-Related Macular Degeneration
Source: Ophthalmol Sci. 2026 Aug 4;6(10):101351. doi: 10.1016/j.xops.2026.101351 (PMC13595137; doi:10.1016/j.xops.2026.101351)
Supplement: Supplementary file 4 [file mmc4.pdf]

Supplementary Table 4

Metric	N	Cases	AUC: age only	AUC: marker only	AUC: age + marker	ΔAUC vs age	Likelihood-ratio P value	Adjusted OR per unit marker
**Control vs AMD**								
Mean sensitivity	96	47	0.768	0.842	0.848	0.081	<0.001	0.21 [0.09–0.50]
AULCSF	94	47	0.763	0.791	0.819	0.056	<0.001	0.34 [0.17–0.68]
CS at 3 cpd	94	47	0.763	0.782	0.816	0.053	<0.001	0.37 [0.20–0.71]
CS at 18 cpd	94	47	0.763	0.760	0.815	0.051	0.005	0.35 [0.16–0.74]
CS at 1.5 cpd	94	47	0.763	0.765	0.811	0.047	0.002	0.39 [0.20–0.75]
Contrast acuity	94	47	0.763	0.758	0.808	0.045	0.010	0.46 [0.25–0.86]
CS at 12 cpd	94	47	0.763	0.757	0.808	0.044	0.003	0.41 [0.21–0.77]
CS at 6 cpd	94	47	0.763	0.763	0.803	0.040	0.002	0.40 [0.21–0.75]
CS at 1 cpd	94	47	0.763	0.740	0.797	0.033	0.012	0.48 [0.26–0.87]
LLVA	96	47	0.768	0.710	0.781	0.013	0.106	0.60 [0.31–1.13]
BCVA	95	46	0.763	0.638	0.766	0.003	0.325	0.77 [0.46–1.29]
**Control vs eAMD**								
CS at 18 cpd	61	14	0.732	0.736	0.788	0.056	0.039	0.33 [0.11–0.98]
CS at 12 cpd	61	14	0.732	0.728	0.781	0.049	0.051	0.42 [0.17–1.04]
AULCSF	61	14	0.732	0.755	0.775	0.043	0.039	0.40 [0.16–1.00]
CS at 3 cpd	61	14	0.732	0.737	0.769	0.037	0.045	0.42 [0.17–1.03]
CS at 6 cpd	61	14	0.732	0.733	0.766	0.034	0.038	0.42 [0.18–0.99]
Contrast acuity	61	14	0.732	0.729	0.763	0.031	0.150	0.52 [0.21–1.30]
LLVA	63	14	0.735	0.687	0.746	0.011	0.263	0.61 [0.25–1.47]

BCVA	63	14	0.735	0.598	0.735	0.000	0.435	0.76 [0.38–1.52]
CS at 1 cpd	61	14	0.732	0.611	0.728	-0.004	0.824	0.91 [0.39–2.13]
CS at 1.5 cpd	61	14	0.732	0.663	0.726	-0.005	0.341	0.66 [0.27–1.59]
Mean sensitivity	63	14	0.735	0.689	0.724	-0.011	0.465	0.67 [0.23–1.98]
**Control vs iAMD**								
Mean sensitivity	82	33	0.781	0.907	0.907	0.125	<0.001	0.08 [0.02–0.27]
CS at 1.5 cpd	80	33	0.777	0.808	0.849	0.072	<0.001	0.29 [0.13–0.62]
CS at 3 cpd	80	33	0.777	0.802	0.836	0.059	<0.001	0.33 [0.16–0.68]
CS at 1 cpd	80	33	0.777	0.795	0.836	0.059	0.001	0.35 [0.17–0.70]
AULCSF	80	33	0.777	0.806	0.835	0.058	<0.001	0.29 [0.13–0.64]
Contrast acuity	80	33	0.777	0.770	0.827	0.050	0.003	0.36 [0.17–0.75]
CS at 18 cpd	80	33	0.777	0.770	0.825	0.048	0.005	0.31 [0.13–0.72]
CS at 12 cpd	80	33	0.777	0.769	0.821	0.044	0.002	0.36 [0.17–0.74]
CS at 6 cpd	80	33	0.777	0.776	0.820	0.043	0.002	0.37 [0.18–0.75]
LLVA	82	33	0.781	0.720	0.796	0.014	0.102	0.56 [0.27–1.14]
BCVA	81	32	0.775	0.655	0.779	0.004	0.287	0.73 [0.41–1.31]
**eAMD vs iAMD**								
Mean sensitivity	47	33	0.584	0.789	0.794	0.210	<0.001	0.16 [0.04–0.60]
CS at 1 cpd	47	33	0.584	0.769	0.771	0.186	0.005	0.28 [0.10–0.77]
CS at 1.5 cpd	47	33	0.584	0.723	0.721	0.136	0.019	0.34 [0.13–0.90]
CS at 3 cpd	47	33	0.584	0.582	0.593	0.009	0.332	0.69 [0.33–1.47]
LLVA	47	33	0.584	0.524	0.590	0.005	0.952	0.97 [0.41–2.30]
CS at 18 cpd	47	33	0.584	0.554	0.589	0.004	0.919	0.95 [0.35–2.55]
BCVA	46	32	0.575	0.540	0.577	0.002	0.872	0.94 [0.46–1.93]

Contrast acuity	47	33	0.584	0.558	0.578	-0.006	0.447	0.76 [0.38–1.54]
CS at 12 cpd	47	33	0.584	0.455	0.578	-0.006	0.653	0.84 [0.40–1.77]
CS at 6 cpd	47	33	0.584	0.442	0.569	-0.015	0.621	0.83 [0.40–1.73]
AULCSF	47	33	0.584	0.435	0.426	-0.158	0.434	0.73 [0.32–1.64]

Supplementary Table 4. Incremental discriminative value of visual function metrics beyond age.

Likelihood-ratio tests compare age-only logistic regression models with nested models including age plus the respective visual function metric. ΔAUC was calculated as the difference between the AUC of the age plus marker model and the age-only model. Odds ratios are derived from age-adjusted logistic regression models and are reported per unit increase in the standardized visual function metric. Results are exploratory and should be interpreted descriptively because several visual function metrics are correlated. AUC, area under the receiver operating characteristic curve.
