# Supplementary material for: Spatial-Frequency–Dependent Contrast Sensitivity in Early and Intermediate Age-Related Macular Degeneration
Source: Ophthalmol Sci. 2026 Aug 4;6(10):101351. doi: 10.1016/j.xops.2026.101351 (PMC13595137; doi:10.1016/j.xops.2026.101351)
Supplement: Supplementary file 5 [file mmc5.pdf]

Supplementary Table 5

Metric	N	Cases	AUC: age + lens	AUC: age + lens + marker	ΔAUC vs age + lens	Likelihood-ratio P value
**Control vs eAMD**						
AULCSF	61	14	0.774	0.807	0.033	0.068
CS at 18 cpd	61	14	0.774	0.804	0.030	0.051
CS at 12 cpd	61	14	0.774	0.802	0.029	0.073
CS at 3 cpd	61	14	0.774	0.796	0.023	0.081
CS at 6 cpd	61	14	0.774	0.792	0.018	0.068
BCVA	63	14	0.776	0.789	0.013	0.490
LLVA	63	14	0.776	0.788	0.012	0.359
Contrast acuity	61	14	0.774	0.786	0.012	0.193
CS at 1.5 cpd	61	14	0.774	0.783	0.009	0.320
Mean sensitivity	63	14	0.776	0.781	0.006	0.824
CS at 1 cpd	61	14	0.774	0.777	0.003	0.645
**Control vs iAMD**						
Mean sensitivity	82	33	0.834	0.924	0.090	<0.001
AULCSF	80	33	0.829	0.886	0.056	<0.001
CS at 1.5 cpd	80	33	0.829	0.879	0.049	<0.001
CS at 3 cpd	80	33	0.829	0.879	0.049	<0.001
CS at 1 cpd	80	33	0.829	0.871	0.042	0.002
CS at 18 cpd	80	33	0.829	0.871	0.042	0.004
Contrast acuity	80	33	0.829	0.870	0.040	0.002
CS at 6 cpd	80	33	0.829	0.869	0.039	0.002
CS at 12 cpd	80	33	0.829	0.868	0.038	0.002
BCVA	81	32	0.828	0.841	0.013	0.202
LLVA	82	33	0.834	0.846	0.012	0.242
**eAMD vs iAMD**						
Mean sensitivity	47	33	0.618	0.807	0.189	<0.001
CS at 1 cpd	47	33	0.618	0.779	0.161	0.006
CS at 1.5 cpd	47	33	0.618	0.751	0.133	0.017
LLVA	47	33	0.618	0.634	0.016	0.823

CS at 3 cpd	47	33	0.618	0.632	0.014	0.299
CS at 18 cpd	47	33	0.618	0.620	0.002	0.997
BCVA	46	32	0.608	0.605	-0.003	0.903
CS at 6 cpd	47	33	0.618	0.608	-0.010	0.620
CS at 12 cpd	47	33	0.618	0.604	-0.014	0.681
AULCSF	47	33	0.618	0.597	-0.021	0.415
Contrast acuity	47	33	0.618	0.589	-0.029	0.449

Supplementary Table 5. Lens-adjusted incremental discriminative value of visual function metrics.

Baseline models included age, lens status, and mild cataract. Full models additionally included the respective visual function metric. ΔAUC was calculated relative to the age- and lens-adjusted baseline model. Likelihood-ratio tests compare nested baseline and full models. Cataract surgery was not included because it was redundant with lens status. AUC, area under the receiver operating characteristic curve.
